# Interleukin 1 receptor antagonist knockout mice show enhanced microglial activation and neuronal damage induced by intracerebroventricular infusion of human β-amyloid

**DOI:** 10.1186/1742-2094-2-15

**Published:** 2005-06-20

**Authors:** Jeffrey M Craft, D Martin Watterson, Emmet Hirsch, Linda J Van Eldik

**Affiliations:** 1 Center for Drug Discovery and Chemical Biology, Northwestern University, Chicago, IL, USA; 2 Cell and Molecular Biology, Northwestern University Feinberg School of Medicine, Chicago, IL, USA; 3 Molecular Pharmacology and Biological Chemistry, Northwestern University Feinberg School of Medicine, Chicago, IL, USA; 4 Obstetrics and Gynecology, Northwestern University Feinberg School of Medicine, Chicago, IL, USA; 5 Department of Obstetrics and Gynecology, Evanston Northwestern Healthcare, Evanston, IL, USA

**Keywords:** Alzheimer's disease, amyloid beta, animal model, glial activation, interleukin-1, microglia

## Abstract

**Background:**

Interleukin 1 (IL-1) is a key mediator of immune responses in health and disease. Although classically the function of IL-1 has been studied in the systemic immune system, research in the past decade has revealed analogous roles in the CNS where the cytokine can contribute to the neuroinflammation and neuropathology seen in a number of neurodegenerative diseases. In Alzheimer's disease (AD), for example, pre-clinical and clinical studies have implicated IL-1 in the progression of a pathologic, glia-mediated pro-inflammatory state in the CNS. The glia-driven neuroinflammation can lead to neuronal damage, which, in turn, stimulates further glia activation, potentially propagating a detrimental cycle that contributes to progression of pathology. A prediction of this neuroinflammation hypothesis is that increased IL-1 signaling *in vivo *would correlate with increased severity of AD-relevant neuroinflammation and neuronal damage.

**Methods:**

To test the hypothesis that increased IL-1 signaling predisposes animals to beta-amyloid (Aβ)-induced damage, we used IL-1 receptor antagonist Knock-Out (IL1raKO) and wild-type (WT) littermate mice in a model that involves intracerebroventricular infusion of human oligomeric Aβ1–42. This model mimics many features of AD, including robust neuroinflammation, Aβ plaques, synaptic damage and neuronal loss in the hippocampus. IL1raKO and WT mice were infused with Aβ for 28 days, sacrificed at 42 days, and hippocampal endpoints analyzed.

**Results:**

IL1raKO mice showed increased vulnerability to Aβ-induced neuropathology relative to their WT counterparts. Specifically, IL1raKO mice exhibited increased mortality, enhanced microglial activation and neuroinflammation, and more pronounced loss of synaptic markers. Interestingly, Aβ-induced astrocyte responses were not significantly different between WT and IL1raKO mice, suggesting that enhanced IL-1 signaling predominately affects microglia.

**Conclusion:**

Our data are consistent with the neuroinflammation hypothesis whereby increased IL-1 signaling in AD enhances glia activation and leads to an augmented neuroinflammatory process that increases the severity of neuropathologic sequelae.

## Background

There is increasing evidence that CNS inflammation (termed neuroinflammation) driven by abnormal or prolonged glia activation contributes to the pathogenesis and progression of both acute and chronic disorders [1,2]. Normally, glia respond to stresses by a transient activation that serves a homeostatic function. However, increased levels of inflammatory and oxidative stress molecules produced by chronically activated glia can lead to neuron damage or death, which can induce further glial activation, thus leading to a self-propagating, detrimental cycle of neuroinflammation and neurodegeneration [3]. A large body of evidence [4-8] suggests that targeting this glia-neuron cycle represents an attractive potential strategy for development of new therapeutic approaches to AD that would alter disease progression. To this end, a more detailed understanding of the proteins, pathways, and inflammatory responses involved in neuroinflammation relevant to AD progression is critical.

One of the biochemical responses of glia to both acute and chronic conditions of brain damage is increased production of the pro-inflammatory cytokine IL-1. An extensive body of research strongly suggests that IL-1 has an integral role in AD pathogenesis and progression. First, analysis of AD brain tissue demonstrates IL-1 overproduction, primarily in the activated microglia that surround β-amyloid (Aβ) plaques and neurons containing neurofibrillary tangles [9,10], the two neuropathological hallmarks of AD. This finding is complemented by the revelation that this overproduction of IL-1 closely corresponds to the level of neuropathology found in a given brain region [11]. Second, cell-based studies show that IL-1 can elicit the production of a number of detrimental molecules from microglia, astrocytes, and neurons. For example, IL-1 can stimulate the production of α 1 anti-chymotrypsin, IL-6, S100B, and inducible nitric oxide synthase [12-15], which are themselves increased in the AD brains [2]. These molecules, either by themselves or by stimulating the production of other molecules, contribute to a neuroinflammatory cascade that has been suggested to result in cell injury, dysfunction, and death in AD[16]. This hypothesis is supported by the neuroprotection observed following suppression of the neuroinflammatory cascade in AD animal models [4,5]. Finally, multiple studies examining IL-1 genetics have shown that polymorphisms in the IL-1gnd IL-1 receptor genes increase the risk of AD by as much as three times in a homozygous carrier [17,16].

All these studies to date are consistent with the hypothesis that increased brain IL-1 levels or activity would correlate with increased severity of AD-relevant neuroinflammation and neuronal damage. To test this hypothesis, we used interleukin-1 receptor antagonist knockout (IL1raKO) mice, which have enhanced IL-1 signaling because of the loss of the IL-1 receptor's physiological antagonist. We induced AD-relevant neuroinflammation and neuronal damage by intracerebroventricular (ICV) infusion of human Aβ1–42 in a mouse experimental model previously developed by us [4,5], and determined the degree of glia activation and neuroinflammation and synaptic degeneration in the hippocampus. We report here that IL1raKO mice are significantly more susceptible than WT mice to the neuroinflammatory and neurodegenerative sequelae of Aβ infusion, supporting the concept that elevated IL-1 signaling in the brain participates in AD pathogenesis.

## Methods

### Interleukin-1 receptor antagonist knockout mice (IL1raKO)

IL1raKO mice were derived as previously described [18] and the colony maintained by mating of heterozygous littermates. Homozygous IL1raKO mice and WT littermates were selected following genotyping, and were allowed to mature until 16 weeks of age before surgery. All mice were kept at the Center for Comparative Medicine (CCM) at Northwestern University Feinberg School of Medicine. All animal procedures were approved by the Animal Care and Use Committee at Northwestern University.

### Aβ infusion

ICV Infusion of human oligomeric Aβ1–42 or vehicle into IL1raKO and WT littermates was performed essentially as described [4]. Briefly, four-month-old mice (n = 5–12 per group) were anesthetized with 2% vaporized isoflurane, and an Alzet micro-osmotic pump (Durect, Model #1002) was attached to a pre-cut 2.5 mm long cannula (Plastics One) stereotaxically implanted into the right lateral cerebral ventricle (at coordinates -1.0 mm mediolateral, -0.5 mm anterioposterior from Bregma; -2.0 mm dorsal-ventral from skull). Pumps contained either vehicle (4 mM Hepes + 250 μg/ml human high-density lipoprotein, HDL) or oligomeric Aβ1–42 (45 μg; American Peptide) [19] dissolved in vehicle. Since HDL normally carries Aβ in serum, it was used in the pump to reduce Aβ aggregation and facilitate better delivery to the neuropil [20,21]. Osmotic pumps were partially coated with paraffin to a point 5 mm above the distal end of the pump. This slows the osmotic passage of water into the pumps' gel casings and has been shown in *ex vivo *experiments to reduce the infusion rate to ~1.6 μg/3.5 μl per day for ~28 days (data not shown).

At 42 days after the start of Aβ infusion, mice were anesthetized with pentobarbital (50 mg/kg) and perfused with a Hepes buffer containing a protease inhibitor cocktail. The superior portion of the cranium was then incised, and brains were removed and longitudinally bisected. In order to exclude the potential that one side of the brain may possess more significant pathology following a unilateral infusion and, therefore, confound the results and/or conclusions, only the *right *half of the brain was fixed in a paraformaldehyde/ phosphate buffer solution and embedded in paraffin for histological examination, while the hippocampus was isolated from only the *left *hemisphere and snap frozen for biochemical evaluation. In addition, the Alzet pumps were examined to insure that the paraffin coating was intact and the reservoir solution was expelled.

### Biochemical analysis of inflammatory and neural markers

Hippocampal soluble extracts were prepared by dounce and sonication in a Hepes buffer containing a protease inhibitor cocktail followed by centrifugation and collection of supernatant as described [4]. Levels of the pro-inflammatory cytokines IL-1β, tumor necrosis factor (TNF)α, S100B, and the presynaptic protein synaptophysin in hippocampal supernatants were determined by ELISA as previously described [4]. Western blots of hippocampal supernatants (10 μg supernatant protein loaded per lane) were done with an antibody to postsynaptic density protein-95 kDa (PSD-95) (1:100,000 dilution; Upstate Biotechnology) as described [4]. Antibodies against β-actin (1:500,000 dilution, Sigma) were used to confirm equal protein loading among the samples. Densitometry was done with ImageQuant software (Molecular Dynamics).

### Histology

Immunohistochemical detection of activated astrocytes and microglia was performed on 10 μm sections with anti-GFAP (1:1500 dilution; Sigma) and anti-F4/80 (1:100 dilution; Serotek) antibodies, respectively, as previously described [4]. Cell counts were determined by two blinded observers and subsequently analyzed as follows. For microglia and astrocyte analysis, all diaminobenzidine (DAB)-stained cell bodies were manually counted in the hippocampus (excluding the fimbria) of three F4/80- and GFAP- labeled sections positioned at -1.8, -2.1, and -2.3 mm from Bregma. In all studies, concordance between observers was within 10% or the section was removed from analysis.

### Statistical analyses

Experimental and control groups were compared as four independent groups using one-way ANOVA with SNK post-hoc analysis using a statistical software package (GraphPad Prism version 4.00, GraphPad Software, San Diego CA, ). GraphPad Prism was also used to construct Kaplan-Meier mortality curves and assess for significance. Statistical significance was assumed when *p *< 0.05.

## Results

### Increased mortality in Aβ-infused IL1raKO mice

**Figure 1:**
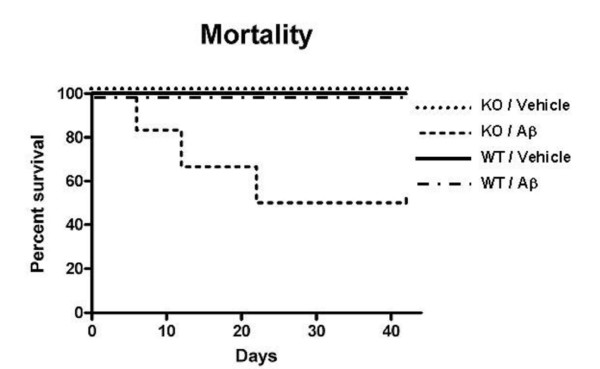
Increased mortality in IL1raKO mice during Aβ infusion. Alzet pumps containing Aβ1–42 or vehicle were surgically implanted in IL1raKO and WT littermate mice (n = 10–12 mice per Aβ-infused group; n = 5 mice per vehicle-infused group), and post-operative survival was monitored for 42 days. Kaplan-Meier survival curves show that WT mice infused with vehicle or Aβ, and IL1raKO mice infused with vehicle experienced no mortality during the time period. In contrast, Aβ-infused IL1raKO mice experienced a 50% mortality rate (6 of the 12 animals died before 42 days). This mortality was significantly different from the other experimental and control groups (error bars = SEM; p < 0.05).

In our previous studies utilizing the Aβ-infusion model [4,5], we found that intra-, peri-, and post-operative animal mortality was approximately 1–2%. Mortality in IL1raKO mice that received an Aβ infusion was much higher, reaching 50% by the time of sacrifice at 42 days (Fig 1). In sharp contrast, no animal mortality was experienced in the IL1raKO mice that received a vehicle infusion, or in WT littermates infused with either Aβ or vehicle.

### Enhanced microglial responses in Aβ-infused IL1raKO mice

**Figure 2:**
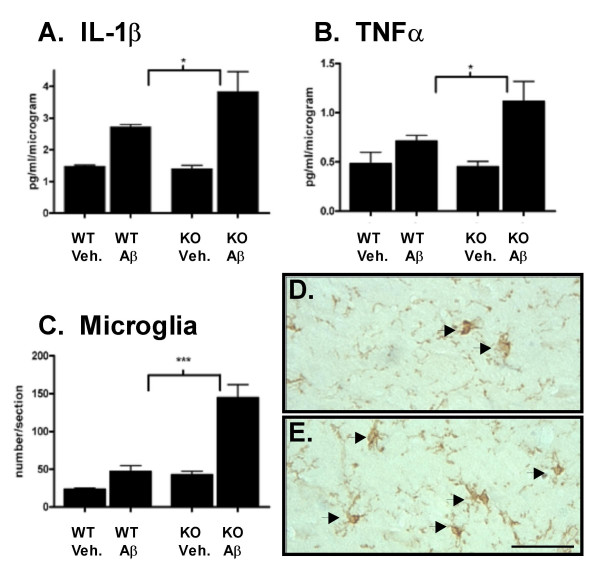
Microglia activation following Aβ infusion. WT and IL1raKO mice infused with Aβ or vehicle for 28 days were sacrificed on day 42 (n = 5–10 mice/group survived for analysis). Brains were bisected and the right side of the brain was processed for immunohistochemistry while the left hippocampus was dissected and used for biochemical analysis. A) Levels of the pro-inflammatory cytokine IL-1β were significantly higher in IL1raKO mice infused with Aβ compared to WT mice infused with Aβ. B) TNFα levels also showed a stronger upregulation in Aβ-infused IL1raKO mice compared to Aβ-infused WT mice. C) F4/80 immunostaining for activated microglia also revealed a significant increase in IL1raKO mice infused with Aβ versus WT mice infused with Aβ. Representative photomicrographs of F4/80-positive microglia in the hippocampus of a D) WT mouse infused with Aβ, and E) IL1raKO mouse infused with Aβ. Arrowheads point to microglia cell bodies. Bar in D-E = 50 μm (error bars = SEM; * Significantly different, p < 0.01; ***Significantly different, p < 0.001).

Based on the high mortality seen in Aβ-infused IL1raKO mice, the infusion experiment was repeated with additional mice to allow survival of enough KO mice for subsequent analyses. At 42 days after the start of surgery, mice were sacrificed and hippocampal tissue analyzed. Measurement of microglia activation endpoints (Fig 2) revealed no significant differences in the basal levels of the pro-inflammatory cytokines IL-1β (Fig 2A) and TNFα (Fig 2B) in vehicle-infused IL1raKO and WT mice. There was a slight increase in the numbers of F4/80 positive microglia in vehicle-infused IL1raKO mice compared with the WT counterparts (Fig 2C). However, in IL1raKO mice infused with Aβ, the intensity of the microglial response, as measured by several biochemical and histological endpoints, was much greater than in Aβ-infused WT mice. For example, IL-1β levels were significantly greater in Aβ-infused IL1raKO compared to Aβ-infused WT mice (Fig 2A). Likewise, TNFα levels were significantly higher following Aβ infusion in IL1raKO mice versus WT littermates (Fig 2B). Finally, the number of activated microglia as measured by F4/80 immunostaining was greater in Aβ-infused IL1raKO mice versus their WT counterparts (Fig 2C). Representative photomicrographs from the hippocampus of WT and IL1raKO mice infused with Aβ (Fig 2D and 2E, respectively) demonstrate the extent of this microglial activation.

### Astrocyte activation in Aβ-infused IL1raKO mice

**Figure 3:**
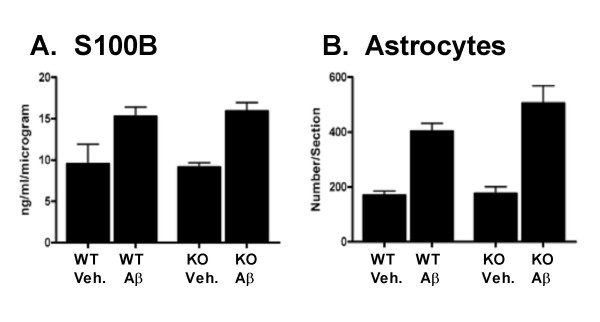
Astrocyte activation following Aβ infusion. WT and IL1raKO mice were infused with Aβ or vehicle, and brains prepared as in Figure 2. A) Levels of the pro-inflammatory astrocyte-derived cytokine S100B showed a similar degree of upregulation in Aβ-infused IL1raKO and WT mice. B) Numbers of GFAP-positive astrocytes were increased to a similar degree in both WT and IL1raKO mice infused with Aβ. (error bars = SEM; p > 0.05 between Aβ-infused IL1raKO and WT mice).

Unlike the findings above with microglia endpoints, we observed no significant differences in astrocyte activation state between IL1raKO and WT mice following Aβ infusion. For example, levels of the astrocyte-derived cytokine S100B were significantly upregulated following Aβ infusion for both the WT and IL1raKO mice (Fig 3A); however, there was no significant difference in the magnitude of this increase between the Aβ-infused WT and IL1raKO mice. These results were also seen by glial fibrillary acidic protein (GFAP) immunohistochemistry. As shown in Fig 3B, there were significant increases in GFAP staining in the hippocampus of all mice following Aβ infusion, and the numbers of GFAP-positive astrocytes were similar in WT and IL1raKO mice.

### Increased synaptic degeneration in Aβ-infused IL1raKO mice

**Figure 4:**
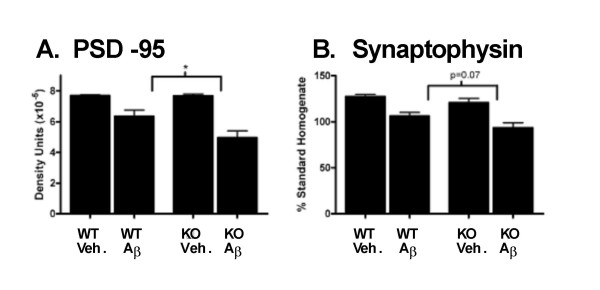
Loss of synaptic markers following Aβ infusion. WT and IL1raKO mice were infused with Aβ or vehicle, and brains prepared as in Figure 2. A) Aβ-infused mice had reduced hippocampal PSD-95 levels compared to vehicle-infused mice, and there was a significantly larger decrease in IL1raKO mice infused with Aβ versus their WT counterparts (error bars = SEM; * p < 0.05). B) The presynaptic marker synaptophysin was reduced in Aβ-infused mice compared to the vehicle-infused mice. In addition, the reduction in synaptophysin in Aβ-infused mice was greater in IL1raKO mice compared to WT mice, although the difference did not quite reach statistical significance (error bars = SEM; p = 0.07).

Given the significant increase in microglial neuroinflammation following Aβ infusion in IL1raKO mice, it was important to investigate the effect of this enhanced neuroinflammation on neuronal responses. Therefore, two different biochemical markers of synaptic degradation were examined. Aβ infusion led to a reduction in levels of the postsynaptic protein PSD-95 in both the IL1raKO and WT mice compared to vehicle-infused mice; however, the reduction was significantly greater in IL1raKO mice compared to WT (Fig 4A). Similarly, levels of the presynaptic protein synaptophysin were reduced in Aβ-infused mice compared to vehicle-infused mice and, while not quite reaching statistical significance (p = 0.07), the reduction in synaptophysin levels was greater in Aβ-infused IL1raKO mice compared to Aβ-infused WT (Fig 4B).

## Discussion

The principle finding of this study is that enhanced IL-1 signaling results in increased mortality, microglial neuroinflammation, and neuronal damage following a chronic infusion of human Aβ1–42 in a murine model. These data provide further support for the idea that IL-1 is an important component in the neuroinflammation cascade that drives AD progression.

An extensive body of evidence indicates the importance of IL-1 in regulating susceptibility to CNS injury. For example, IL-1β levels in cerebrospinal fluid (CSF) are substantially increased shortly after severe traumatic brain injury in humans, and the magnitude of this increase is directly proportional to intracranial pressure [22]. Animal studies have also demonstrated the importance of IL-1 in mediating damage following both an acute insult, such as neonatal hypoxia-ischemia [23], and the progressive neurodegeneration that follows mild acute insults in rodents [24]. This is not unexpected given the array of potentially detrimental molecules produced by the CNS in response to increased production of IL-1. For example, IL-1β and/or IL-1α have been implicated in the production of other pro-inflammatory cytokines such as S100B [14]. Furthermore, IL-1β can stimulate glial iNOS production [15], which in turn can greatly increase the oxidative stress experienced by the brain and potentially lead to neuronal damage through protein nitration pathways [25].

More relevant to the current study, there is increased IL-1 signaling in chronic neurodegenerative diseases. In addition to the IL-1 overexpression and disease-relevant distribution in AD [26,10], IL-1 is also increased in other chronic conditions that involve neurodegeneration. These include Down's syndrome [26], which possesses many of the neuropathological hallmarks of AD, Creutzfeldt-Jakob disease [27], and HIV dementia [28]. In particular, *in vivo *rodent models of AD have also revealed a correlation between the extent of neuropathology and the level of IL-1 production [4,5,21]. Most importantly, a number of different therapeutic interventions targeted towards decreasing neuroinflammation have been shown to both decrease IL-1 production and reduce the amount of synaptic degeneration and neuron death [8,4,6]. These observations support the utility of measuring IL-1β levels, in terms of demonstrating a linkage to disease progression and monitoring response to therapeutic interventions that result in attenuation of disease.

The results of the current study, in which a rodent model that has increased IL-1 signaling due to loss of the IL-1 receptor's physiologic antagonist shows enhanced Aβ-induced neuroinflammation and neuronal damage, are consistent with previous work in the field. The increases in TNFα levels and F4/80-positive cells document that enhanced IL-1 signaling stimulates a robust and generalized microglia response following Aβ infusion. These observations also illustrate the escalating, cyclical nature of the Aβ-induced neuroinflammatory response, since with enhanced IL-1 signaling there are also increased levels of IL-1β itself. This is similar to findings with the IL1raKO mouse in models of systemic inflammation [18]. In addition, the resultant increased neuroinflammation in the IL1raKO mice infused with Aβ was accompanied by an exacerbation in the loss of synaptic markers, especially PSD-95. This particular finding, in conjunction with our similar findings in Aβ-infused S100B overexpressing transgenic mice [29], strongly argues for the conclusion that animals predisposed to neuroinflammation suffer more severely from neurodegenerative sequelae following Aβ infusion. Evidence from the epidemiological assessment of AD risk factors also supports this conclusion. Previous head injury, for example, is a significant environmental risk factor for development of AD in which it is hypothesized that IL-1-mediated neuroinflammation plays a key role [30,31].

A somewhat surprising finding was that, unlike the enhanced microglia and neuronal responses in the Aβ-infused IL1raKO mice compared to WT mice, the astrocyte responses to Aβ infusion were very similar in the two mouse strains. Both IL1raKO and WT mice showed similar upregulation of S100B levels and GFAP immunoreactivity after Aβ infusion. A possible explanation is that, at the time point examined (42 days), astrocyte responses had not yet reached their maximum following Aβ infusion. This possibility indicates a need for future studies to examine the temporal development of microglia, astrocyte, and neuronal responses after start of Aβ infusion.

The IL1raKO mice infused with Aβ experienced extensive mortality during the course of the experiment, despite minimal mortality of other strains of mice in our previous studies [4,5,29]. At first inspection, this increased mortality could be explained by the pro-inflammatory status of IL1raKO mice, which may predispose them to systemic septic-like episodes at a higher frequency than their WT littermates, especially following a major surgical operation to place an indwelling pump and ICV catheter. However, the lack of mortality in the IL1raKO mice that received a vehicle infusion would argue against this conclusion. A more intriguing possibility is that these mice died either directly or indirectly from a more severe neuroinflammatory response to Aβ than the mice that survived. The robust and consistent neuroinflammation, which is one of the key hallmarks that characterizes the Aβ infusion model, supports this conclusion as a distinct possibility. While quite interesting, especially in light of a similar syndrome afflicting a subset of individuals enrolled in the now discontinued Aβ vaccine trials [32], elucidation of the mechanisms underlying the increased mortality will require additional research.

## Conclusion

The major finding of this study is the demonstration that IL1raKO mice show selective up-regulation of microglial neuroinflammation and increased neuronal damage following Aβ infusion when compared to WT littermates. The susceptibility of the IL1raKO mice to increased Aβ-induced neuroinflammation was demonstrated by biochemical and histological measurements of microglial activation. This increase in microglial activation in the IL1raKO mice is also associated with an increase in the degree of synaptic degeneration observed following Aβ infusion, suggesting that enhanced IL1 signaling leads to deleterious neuroinflammation that either directly damages neurons and/or potentiates the neurotoxic effects of Aβ. These data provide further support for the hypothesis that increases in the level of IL1 signaling in the AD brain can be detrimental through the cytokine's role as a key component of the neuroinflammatory cascade that contributes to progression of neuropathology. It also suggests that manipulation of IL-1 signaling and other neuroinflammatory mediators and pathways could be utilized to develop clinically meaningful, disease-modifying AD therapies.

## Competing interests

The authors declare that they have no competing interests in the outcome, results, or conclusions of these studies.

## Authors' contributions

JMC helped conceive the study and conducted animal surgeries, care, and biochemical/ histological assays. DMW helped conceive the study, interpret the results, and assist in the preparation of the manuscript. EH developed and provided the IL1raKO mice and gave helpful advice for handling and care of the animals. LVE helped conceive the study, analyze data and assist in the preparation of the manuscript.
